# Identifying the conditions needed for integrated knowledge translation (IKT) in health care organizations: qualitative interviews with researchers and research users

**DOI:** 10.1186/s12913-016-1533-0

**Published:** 2016-07-12

**Authors:** Anna R. Gagliardi, Mark J. Dobrow

**Affiliations:** Toronto General Hospital Research Institute, University Health Network, Toronto, Canada; Institute of Health Policy, Management & Evaluation, University of Toronto, Toronto, Canada

**Keywords:** Implementation science, Health services research, Collaboration, Integrated knowledge translation, Qualitative research

## Abstract

**Background:**

Collaboration among researchers and research users, or integrated knowledge translation (IKT), enhances the relevance and uptake of evidence into policy and practice. However, it is not widely practiced and, even when well-resourced, desired impacts may not be achieved. Given that large-scale investment is not the norm, further research is needed to identify how IKT can be optimized.

**Methods:**

Interviews were conducted with researchers and research users (clinicians, managers) in a health care delivery (HCDO) and health care monitoring (HCMO) organization that differed in size and infrastructure, and were IKT-naïve. Basic qualitative description was used. Participants were asked about IKT activities and challenges, and recommendations for optimizing IKT. Data were analysed inductively using constant comparative technique.

**Results:**

Forty-three interviews were conducted (28 HCDO, 15 HCMO) with 13 researchers, 8 clinicians, and 22 managers. Little to no IKT took place. Participants articulated similar challenges and recommendations revealing that a considerable number of changes were needed at the organizational, professional and individual levels. Given the IKT-absent state of participating organizations, this research identified a core set of conditions which must be addressed to prepare an environment conducive to IKT. These conditions were compiled into a framework by which organizations can plan for, or evaluate their capacity for IKT.

**Conclusions:**

The IKT capacity framework is relevant for organizations in which there is no current IKT activity. Use of the IKT framework may result in more organizations that are ready to initiate and establish IKT, perhaps ultimately leading to more, and higher-quality collaboration for health system innovation. Further research is needed to confirm these findings in other organizations not yet resourced for, or undertaking IKT, and to explore the resource implications and mechanisms for establishing the conditions identified here as essential to preparing for IKT.

**Electronic supplementary material:**

The online version of this article (doi:10.1186/s12913-016-1533-0) contains supplementary material, which is available to authorized users.

## Background

Population-based studies demonstrate that healthcare delivery and outcomes often vary from recommendations that are based on the best available evidence [1–3]. Implementation science investigates why such circumstances exist, and how to promote the use of evidence in health care decision-making [4]. It draws upon an array of disciplines, necessitating collaboration among individuals with different expertise (theoretical, professional, methodological) and roles (researchers, research users) to fully understand how complex health care problems can be addressed. Collaboration among researchers and research users (clinicians, managers, policymakers, health care users) is not a new concept. Others have recognized that engaged scholarship blends the different perspectives of researchers and decision-makers such that the knowledge produced is relevant to real world problems [5]. This co-production of knowledge is now more commonly referred to as integrated knowledge translation (IKT), and defined as an ongoing relationship between researchers and research users for the purpose of collaboratively engaging in a mutually beneficial research project or programme of research to support decision-making [6].

IKT has been shown to improve the uptake of evidence into policy and practice [7]. It also contributes to numerous intermediate outcomes. Collaboration between researchers and users reveals differing perspectives, expectations and values to create trust and a shared vision that enable on-going and effective partnership, thereby contributing to the capacity for IKT [8]. On a practical level, users can inform research questions that are relevant to practice or policy; refine research methods and/or data analysis; synthesize findings; and disseminate or implement findings or products [9]. Users benefit from interaction with researchers through a broadened reflection on their own activities, enhanced knowledge and skills, information about other pertinent research, and new contacts with other researchers or users [10].

However, it appears that IKT is challenging to initiate and sustain. Reported barriers include lack of knowledge, resources or incentives for IKT [11–14]. As a result, IKT is not routinely practised. A survey of 240 Canadian health services researchers, and another survey of 265 directors of Canadian research organizations found that the majority did not engage in IKT [11, 15]. Similarly, a survey of health policy experts representing 30 European countries found that there were no explicit IKT mechanisms in most respondent countries [16]. The need to foster IKT was recognized in both nursing and primary care sectors in Australia and the United Kingdom; among emergency medicine professionals from 16 countries; and by representatives of 33 research funding agencies in Canada, Australia, France, the Netherlands, Scandinavia, the United Kingdom, and United States [17–22].

There is little empirical research on how to undertake IKT. Interviews with participants of seven research programs revealed four stages in which research users were most actively involved - conceptualization, data collection, interpretation and dissemination [10]. In each of these stages engagement varied - research users commonly supported the research in principle but were often not directly involved as integral partner. Two national-level initiatives that invested heavily in IKT are notable. Collaborations for Leadership in Applied Health Research and Care (CLAHRC) were funded in the United Kingdom to encourage regional interaction and collaboration among researchers and research users, and improve local health service use and outcomes [23]. While the number of collaborative research studies increased, interviews with 174 participants across nine CLAHRCs and in-depth case study of four CLAHRCs revealed that most were based on pre-existing relationships and patterns of interaction remained the same, therefore goal-setting and activities were largely investigator-driven, with little impact on health services and outcomes [24]. The Netherlands established nine Academic Collaborative Centres (ACC) for Public Health [25, 26]. Evaluation of one ACC similarly revealed that the number of projects increased but were largely academically-focused and wide-spread improvements were not realized [27].

It appears that even under favourable resource conditions, IKT is challenging and desirable outcomes are not easily achieved. Such large-scale investment is not widely replicable in jurisdictions with health care funding constraints therefore further research is needed to generate insight on how IKT can be optimized. Some direction for further study was revealed by Ward et al. in a case study of three health service delivery programs which found that IKT was dynamic and highly influenced by the setting within which decisions were made [28]. Rather than imposing external or rigid IKT structures and processes, the authors of that work suggested that IKT could be optimized by first examining naturalistic interaction that takes place in a given program or organization to identify how IKT could be tailored and enhanced for that setting, particularly given limitations in resources or capacity. Therefore the purpose of this study was to explore IKT in different settings in which there was no pre-existing program or other investment in IKT to describe baseline IKT activities and how they could be optimized. A secondary purpose was to use insight from the naturalistic assessment to generate a framework by which other organizations could plan or assess capacity for IKT.

## Methods

### Approach

As there is limited knowledge about how to optimize the conduct and impact of IKT, qualitative methods were employed [29]. Interviews were conducted with researchers and research users in two organizations. Given that we wished to describe factual information about factors influencing IKT processes rather than generate theory, basic qualitative description was employed [30]. This approach recognizes that straightforward accounts are often needed and, the methods of collecting and analysing data similar to those used for other qualitative approaches [29]. Rigour was optimized by sampling participants with various characteristics to explore potentially different views; analyzing responses inductively so that findings emerged from the data; comparison of independently-derived analyses to enhance trustworthiness of the findings, and member-checking to ensure that participants views were accurately captured [31]. We complied with Consolidated criteria for reporting qualitative research (COREQ) reporting standards (Additional file 1) [32]. The investigators had no relationship with the participants.

### Sampling and recruitment

Two organizations accessible to the investigators that both produced and used health services research evidence, and in which there was no formal program to stimulate IKT, were purposively chosen to differ by type of organization, size and research infrastructure as these factors could influence whether and how IKT was practised. The health care delivery organization (HCDO) was comprised of several hospitals, provided services to a large urban and connected regional population, and employed 12,000 staff. Research was formally supported through several research institutes. The health care monitoring organization (HCMO) employed 1,000 staff, advised government on the funding, operation and outcomes of screening, prevention and treatment programs, implemented quality standards, and measured and reported on health system performance. Research was coordinated through the position of a Director but not centralized in a research institute.

Purposive sampling was primarily used to recruit health services researchers, and various types of research users including clinicians and managers in different departments or programs in both organizations. Participants were identified through analysis of organizational web sites and publicly available documents. Purposive sampling was supplemented with snowball sampling, where additional participants were identified by referrals from interviewees. Participants were not selected according to pre-assessed knowledge or experience of IKT. By email invitation they were invited to take part in a single interview. Non-responders were contacted by email at 2 and 4 weeks, and by telephone at 6 weeks. We aimed to interview five researchers and five research users (clinicians, managers) from each of two organizations for a minimum target of 20 interviews. Sampling was concurrent with data collection and analysis, and proceeded until informational redundancy, or no further unique themes emerged with successive interviews. This was determined by prospective review of transcripts and discussion among the investigators.

### Data collection

An interview guide was developed and pilot tested through an interview with one researcher and one manager to refine wording and flow of questions (Additional file 2). To establish mutual understanding the interviewer defined IKT as instances of collaboration among researchers, clinicians, managers and policy-makers to generate and/or apply research. Each participant was asked to describe their professional role to understand whether they responded primarily from the perspective of researchers or research users, how they were involved in IKT, and its purpose or impact. To understand how IKT could be enhanced or tailored, participants were asked to describe factors that enabled or challenged IKT, and how IKT could be supported. Interviews were conducted in-person or by telephone depending on participant preference, and audio-recorded. The principal investigator conducted HCDO interviews (October 2010 to February 2011), and trained and coached a research assistant who conducted HCMO interviews (February to May 2012).

### Data analysis

Transcripts were examined using constant comparative technique to inductively identify unique themes [29]. To establish an initial coding framework of relevant themes, the principal investigator and research assistant independently read several interview transcripts, and identified, defined and organized themes that emerged from the data (first level coding). The two met to review themes and resolve discrepancies through discussion. The research assistant proceeded to analyse all transcripts to identify all instances of themes in the coding framework, and expanded or merged thematic codes (second level coding). The principal investigator reviewed all data analysis independently. Data were tabulated and compared by theme, organization and professional role. Member checking was undertaken by distributing a summary report to participants who were invited to provide feedback as a means of confirming the findings. No responses were received. Recommendations for promoting and enabling IKT that emerged from this research were compiled into a framework by which organizations can plan or assess IKT capacity at the organizational, professional and individual level. Relational analysis was used to do this [33]. In this technique, all data is perused and each unique finding is tallied. For this study, each instance of a unique recommendation for supporting IKT offered by participants was tallied, and then compiled into the framework.

## Results

### Participants

A total of 43 interviews were conducted with researchers, clinicians, and managers in the HCDO (28 of 54 invited) and HCMO (15 of 20 invited) organizations (Table 1). Key findings, which were similar within and across organizations, are summarized in tables and discussed here.

**Table 1:** Interview participants

Primary profession	Participants (n)		Total
	Delivery organization	Monitoring organization	
Researchers	7	6	13
Clinicians	5	3	8
Managers/Directors	16	6	22
Total	28	15	43

### IKT not practiced but desirable

While participants from both organizations were engaged in generating and using a broad spectrum of academic research aimed at improving health services and outcomes, they stated that IKT was not used to do so. Researchers and research users in both organizations said that they did not typically interact with individuals representing other professions for planning, undertaking or implementing health services research.

Most research is done in silos and is grant-based, mostly clinicians and researchers. I don’t think there’s a lot of interaction with people who run the hospital [HCDO Director 26].

I don’t have a lot of interaction with [managers] other than filing a report once a year [HCMO Researcher 34].

However, participants from both organizations said that efforts were needed to connect researchers and researcher users, therefore IKT was viewed as desirable and useful.

You need to put in place structures that allow people to overcome those tribal boundaries….set up social institutions that break those barriers down [HCDO Researcher 07].

It is unacceptable that a health services researcher would go off and just study an event without understanding the clinical issues. And in the same way, we shouldn’t be studying the clinical issues independently of the health services researchers. The two parallel processes need to be married much more closely [HCMO Clinician 35].

### Strategies to enhance IKT

Table 2 shows the numerous IKT challenges articulated by participants, and their corresponding recommendations to promote and support IKT which were categorized as organizational, professional and individual level issues and strategies. Select quotes are included here to illustrate key points. There was no single over-riding recommendation suggesting that considerable changes were needed across all three categories to support IKT. Although participants represented organizations that differed by type, size and research infrastructure, the experiences and recommendations of researchers and research users in both organizations were similar, suggesting that, when IKT is absent, a core set of conditions may need to be established in any setting before IKT can take hold and germinate.

**Table 2:** Exemplar quotes representing IKT challenges and recommendations expressed by interview participants

Theme	Challenge	Recommendation
Organizational	*Lack of infrastructure to support collaboration* • There is no mechanism at the moment to foster interaction other than the personal contacts we have [HCDO Researcher 03] • The researchers are out there working on their own in isolation [HCMO Director 43] • We don’t have IT systems that work and talk to each other [HCDO Clinician 23] • One thing that people have been very frustrated about is that they have not been able to share their data with others for research [HCMO Researcher 31]	*Philosophy of collaboration* • Recognition from the highest levels of the organization…identify this as an area of work that is valuable [HCDO Researcher 01] • Creating an environment where these groups can thrive and work together [HCDO Clinician 22] *Infrastructure and dedicated resources* • Health services research needs to actually increase their infrastructure [HCMO Clinician 35] • There needs to be a concerted effort to put resources behind it [HCDO Clinician Researcher 23] *Align IKT with organizational priorities* • Identify what health services research issues are going to be priorities [HCDO Clinician Research 12] • We should be directing in a more clear way with a goal in mind to health services research rather than the health services research kind of doing fishing expeditions [HCMO Manager 35]
Professional	*Lack of research expertise* • We lack of lot of expertise that would be helpful for health services like biostats, health economists, sociology, psychology [HCDO Researcher 06] *Differing cultures between researchers and managers* • The culture of management and the culture of research are different [HCDO Researcher 07] • So it’s just different cultures [HCMO Researcher 30] *Silos of clinicians and types of researchers* • Organizational structure is still driven by the silos of medicine, nursing, allied health [HCDO Clinician 25] • It may also be a need to work not in silos so the people who have the data won’t be isolated from the people who need the data [HCMO Researcher 30] *Unclear who bears responsibility for initiating collaboration* • Research has to make more of an effort to … make known what could be available to the clinician [HCMO Manager 40] *Not aware of who to contact* • I don’t think I…we have a way of having a good picture of who’s even out there [HCMO Director 43] • They often don’t know that each other exits [HCDO Researcher 04]	*Accrue a critical mass of researchers with differing expertise* • Creating a critical mass of researchers [HCDO Clinician 21] • We’re gonna need to invest in people [HCMO Researcher 30] *Engage intermediaries to broker partnerships* • You need knowledge brokers…who you would contact and say I’m interested in looking at whatever…who’s the policy maker looking at that? [HCDO Clinician 11] *Embed researchers within clinical or management units* • [researchers should] come out of your office, appear on the ward, go to the rounds, sit in the clinic, talk with doctors and nurses and pharmacists [HCDO Manager 19] *Develop a directory of research/researchers* • Some kind of directory…to find people who are content experts and some of their ongoing research to get a flavour of who I might want to approach [HCDO Clinician 11] • Some kind of interactive forum for lodging research questions that feed out to a broader user community [HCDO Researcher 04] *Create forums to enable collaboration* • The managers come present some of their challenges and concerns…or the researchers present their work [HCDO Clinician 11] • Clinical experts and health services researchers all at the same table would be a useful thing [HCMO Clinician 35]
Individual	*Lack of familiarity or comfort with IKT* • [Researchers are] not well embedded into our processes and we’re not well embedded into theirs [HCMO Director 43] • Their goals may be very similar but they may not really know how to talk to each other [HCDO Researcher 02] *Time required for IKT* • I don’t know how we maintain the relationships because you know how busy people are [HCMO Researcher 30] • Everyone’s extremely busy so getting together isn’t going to happen [HCDO Manager 17] *Lack of incentives or accountability mechanisms* • There really hasn’t been any relationship between management and researchers which is understandable because incentives, experience and accountability differ [HCDO Researcher 07]	*Provide IKT training* • Hold some workshops around how to actually do this to develop that whole skill set [HCDO Manager 09] *Incentivize IKT with accountability mechanisms and recognition* • If health services researchers got credit for impact at a system level, it would influence the way in which they select topics, put together the research teams and knowledge transfer strategies [HCMO Manager 39] • The measure of success may not be publications, it could be developing practice innovations, a new model of care, cost efficiencies, improved access for patients, new educational programs [HCDO Manager 21] • Incentives to recognize people for their time…stimulate people’s interest and appeal to what they’re doing and make it look worthwhile [HCDO Clinician 29]

*HCDO* health care delivery organization, *HCMO* health care monitoring organization

#### Organizational level

At the organizational level, infrastructure was lacking to support IKT. To address this, participants recommended that organizations allocate sufficient and dedicated resources that would enable IKT. This included a designated leader, coordinators, a coordinating office, and meeting space.

It wouldn’t happen organically…there would have to be some manipulation of people and their priorities…a director and staff that could actually facilitate this sort of thing [HCDO Clinician 14].

Apart from dedicated resources, participants noted the overarching importance of establishing an organizational philosophy of collaboration by which to jointly generate research and to encourage its use in decision-making. If the organization was overtly committed, this would establish value for IKT, overcome the differences in culture between researchers and decision-makers that limited interaction, and establish new social norms that included collaboration.

[IKT] has got to be placed as one of the values [HCMO Researcher 30].

As a way to enhance its perceived value and promote its practice, it was suggested that IKT activities and resources be aligned with organizational priorities.

It should be research that would benefit the organization and not be a stand-alone academic issue [HCDO Clinician Manager 21].

#### Professional level

At the professional level participants spoke of silos and of differing philosophies between researchers and research users and, within professions, between those with differing specialties. They also noted that the responsibility for initiating partnerships was unclear. To address these challenges participants suggested that intermediaries could broker partnerships and assist with communication between those partners. They also recommended that positions be embedded in clinical or management departments/units as this would prompt both formal and informal communication, leading to collaboration.

You could have people who function as matchmakers….to help people talk the same language [HCDO Researcher 04].

Align business managers with clinical programs [HCDO Manager 20].

Should they feel compelled to do so, participants said they would not know who to contact. Recommendations that addressed this challenge included a directory of research/researchers, and forums at which researchers and research users could interact to stimulate collaboration.

Getting an inventory of what’s going on in the organization [HCDO Manager].

Clinical experts and health services researchers all at the same table would be a useful thing [HCMO Clinician 35].

At the professional level was a lack of researchers with whom managers could engage in collaborations so participants expressed the need for a critical mass of researchers with differing expertise to be employed or somehow affiliated with the organization.

You need to have infrastructure and for this kind of research it’s human equipment [HCDO Clinician 24].

#### Individual level

Various challenges impeded IKT at the individual level including lack of familiarity or comfort with IKT, lack of time, and a lack of incentives for motivating time spent on IKT. Recommendations to address these challenges included training in IKT, and formal recognition of time spent on IKT and associated achievements.

I think that if health services researchers got credit for impact at a system level, it would influence the way in which they select topics, put together the research teams and their knowledge transfer strategies [HCMO Manager 39].

### IKT planning/evaluation framework

All unique recommendations for supporting IKT revealed by this research were integrated in a framework that can be used by organizations to plan, or assess their capacity for IKT at the organizational, professional and individual levels (Table 3).

**Table 3:** Proposed components of a framework to assess organizational capacity for IKT

Component of IKT capacity	Conditions conducive to IKT capacity
ORGANIZATIONAL	
Culture or philosophy of IKT	• The organizational culture is seen to promote and foster IKT • IKT is recognized by the organization at the highest levels in its goals, strategic plans, performance measures, and operational budget, and advocated by senior leadership • The organization actively promotes collaboration across departments or units
Dedicated resources to support IKT	• Dedicated resources are allocated for IKT including leaders, coordinators, space, forums and information systems
IKT linked to organizational priorities	• IKT resources and activities are linked with organizational goals
PROFESSIONAL	
Identifying collaborators and initiating IKT	• Staff members are aware of individuals for the purpose of collaboration, and how to identify them • A directory is in place by which to identify researchers or research users for the purpose of collaboration • Staff members are empowered to take the responsibility for initiating collaboration
Linkages are facilitated by brokers or embedded positions	• Intermediaries or facilitators are in place specifically to support IKT • Researchers are embedded in departments or units • Researchers and research users are familiar with each other’s HSPR needs and values
Critical volume of researchers	• Expertise is in place or available, including scientists with knowledge and skill in various disciplines and research methods
Forums offer opportunities for interaction	• A variety of forums, both in-person and technology-enabled are in place to support interaction that may give rise to, or enables IKT • Researchers and research users initiate, lead and participate in IKT forums
INDIVIDUAL	
IKT skill or knowledge	• Staff are familiar with the concept of, and approaches for IKT • Education and training are in place or available to develop value and skills for IKT among staff of all levels
Time for IKT	• Time for IKT is accommodated or scheduled
IKT is incentivized and recognized	• Staff members are accountable for IKT activities • Time spent on, and the outcome or impact of IKT activities are recognized in performance reviews

## Discussion

This study explored IKT infrastructure and activities in two organizations that differed according to type, size and research infrastructure. Despite differences in organizational characteristics, similar views and experiences were articulated within and across organizations. Health services researchers and research users in both organizations said that little IKT took place. Both organizations generated or used research in traditional ways characterized by little or no inter-professional collaboration. This could be referred to as an IKT-absent state, distinguished from one where IKT was established but early and still developing, or more well-established. Participants articulated similar challenges and recommendations revealing that a considerable number of changes were needed at the organizational, professional and individual levels. Given the IKT-absent state of participating organizations, this research identified a core set of conditions which must be addressed to prepare an environment conducive to IKT. These conditions were compiled into a framework by which organizations can plan for, or evaluate their capacity for IKT.

This study was unique from other studies that evaluated IKT [11–14] because the findings are relevant to organizations in which IKT is absent and in which there is a need to establish basic and fundamental conditions so that IKT can take root. In comparison, other research has explored IKT in organizations where it was already resourced and/or introduced, therefore IKT challenges and corresponding recommendations differed from those particular to the IKT-absent organizations that participated in this study [10, 25–28]. This is also true of more recently published research. For example, a survey of, and interviews with researchers and research users already engaged in IKT revealed that role clarity, changes in partners due to organizational turnover, limited communication about goals and differing timelines challenged IKT although equal rather than token partnerships were better able to manage these challenges [34]. Survey of, and interviews with researchers and research users in a two-year transnational IKT partnership revealed differences in participant satisfaction with the use of a common language, communication about roles and expectations, and frequency of communication about project progress [35]. Similarly, a recently published systematic review of IKT in rehabilitation research found that factors influencing already-established partnerships included upfront negotiation of roles and expectations, adaptation of the scientific language of research materials, and power-sharing of the planning and research process [36]. A literature review and interviews with researchers and research users affiliated with the United States Veterans Health Administration and the United Kingdom National Health Service CLAHRCs generated a matrix by which to establish responsibilities and tasks among those taking part in established IKT partnerships [37]. Our research is also distinct from that of Guise et al. who, through literature review and interviews, generated a checklist of steps for negotiating research priorities with stakeholders [38], and from that of Kothari et al. who generated indicators of researcher-policymaker collaborations which largely reflect the impact or outcomes of IKT rather than the philosophy and infrastructure that must be in place to support it [39].

The output of this research is therefore novel and two-fold. It reinforces the concept, originally proposed by Ward et al. [12] of planning or evaluating IKT based on naturalistic assessment to categorize an organization’s IKT maturity or readiness for IKT. It also generated a framework by which organizations in which little or no IKT takes place can assess their readiness for IKT and plan for the capacity to enable IKT. This framework can be used by health services researchers to anticipate the challenges they may face in establishing partnerships, by managers and policy-makers when allocating resources, and by research funders when assessing the comprehensiveness and feasibility of IKT plans. The framework could also serve as the basis for future work to develop quantitative measures of IKT readiness, and build on work by Kothari et al. that validated a tool to examine health organization capacity to use research which included a few questions relevant to interaction among researchers and researcher users [40].

Researchers and research users from both organizations said that an IKT philosophy or culture must be established. This included high-level promotion of the idea and value of IKT, organizational commitment to enable IKT through dedicated resources, and incentives to motivate or recognize IKT and its impact. This represents a fundamental and profound change in the way that research is conducted from the traditional investigator-driven model to one where multiple stakeholders participate in joint knowledge production. Therefore further research is needed to develop, implement and evaluate IKT approaches and interventions, and demonstrate their impact on research, and on health care planning, delivery and outcomes. Such evidence may be needed to both convince stakeholders that IKT is important, and to generate objective guidance on how to undertake and achieve IKT. As a start, it may be useful to explore the resource implications and mechanisms for establishing the conditions identified here as essential to preparing for IKT. There has been little use of theory in studies evaluating IKT therefore future research could be informed by a variety of theories and conceptual frameworks for interprofessional collaboration such as those identified in a systematic review of 27 studies by D’Amour et al. [41].

The IKT capacity framework generated in this study must be elaborated and validated through future research. Participants identified the need for dedicated leadership or a coordinator role that would enable partner identification and interaction for IKT. The facilitator role is recognized by a variety of terms including opinion leaders, champions, knowledge brokers, and linking agents [42]. The impact of such roles on research use and impact has been variable [43, 44] and it is not clear how best to choose, train and operationalize such a role. However, it appears that multiple roles may be essential. For example, authoritative opinion leaders or organizations may be needed to promote the philosophy of IKT, local champions may be needed to model the behaviour, and dedicated facilitators may be needed to support IKT efforts. Guidance on how to promote and enable IKT could also be drawn from the mentoring literature which describes a variety of strategies for work-situated training and support of professional activities [45, 46]. Further research is needed to establish optimal roles and processes for various types of IKT-enabling agents.

Interpretation and application of these findings may be limited in that we explored IKT in two organizations only in a single jurisdiction with a publicly-funded health care system. Similar studies are needed in other settings to confirm the relevance of these findings. Furthermore, the findings are relevant to IKT-absent organizations and may not be useful to organizations where IKT is already practiced that are faced with different types of challenges. However, our research was meant to be exploratory in nature, and rigorous methods were used to explore the views of multiple stakeholders including researchers, clinicians and managers across two organizations with different characteristics.

## Conclusions

This study revealed a core set of conditions which may need to be established in organizations with little or no IKT activity to create a fertile environment for IKT. A considerable number of conditions were identified at the organizational, professional and individual levels. These conditions were compiled into a framework by which organizations can plan for, or evaluate their capacity for IKT. Use of the IKT framework may result in more organizations that are ready to initiate and establish IKT, perhaps ultimately leading to more, and higher-quality collaboration for health system innovation. Further research is needed to confirm these findings in other organizations not yet resourced for, or undertaking IKT, and to explore the resource implications and mechanisms for establishing the conditions identified here as essential to preparing for IKT.

## Abbreviations

ACC, academic collaborative centres for public health; CLAHRC, collaborations for leadership in applied health research and care; HCDO, health care delivery organization; HCMO, health care monitoring organization; IKT, integrated knowledge translation

## Additional files

Additional file 1: Consolidated criteria for reporting qualitative research (COREQ) reporting standards. (DOC 56 kb) Additional file 2: Interview guide. (DOC 27 kb)
