# Supplementary material for: Identifying the conditions needed for integrated knowledge translation (IKT) in health care organizations: qualitative interviews with researchers and research users
Source: BMC Health Serv Res. 2016 Jul 12;16:256. doi: 10.1186/s12913-016-1533-0 (PMC4943023; doi:10.1186/s12913-016-1533-0)
Supplement: Additional file 2: — Interview guide. (DOC 27 kb) [file 12913_2016_1533_MOESM2_ESM.doc]

**Additional File 2. Interview guide**

**PROFESSIONAL ROLE**

Please describe your professional role

Prompt

Establish whether researcher, clinician, manager or combination of these

**UNDERSTANDING**

How would you define integrated knowledge translation?

Prompt

If needed, clarify that IKT refers to collaboration among researchers, clinicians, managers, policy-makers and possibly other types of research users to generate and/or apply research.

**IMPACT**

What do you feel IKT achieves?

Prompt

How does IKT contribute to different outcomes? What are the impacts?

**CURRENT MODEL**

Briefly describe whether and how you are involved in IKT, either for your own research, or in collaboration with researchers.

Prompt

- What type of professionals were/are involved?
- How do they interact (type of forum, frequency, purpose)
- For what activities do they interact (coming up with research questions, recruitment, collecting or analyzing or interpreting data, disseminating or implementing the findings)?

**ENABLERS**

How does your organization currently enable IKT?

Prompt

release time, space, forums, admin support, training, mentoring, leadership, incentives, other

**CHALLENGES**

What factors challenge your participation in IKT?

Prompt

Knowledge/skill, attitude, awareness of individuals to fill specific roles, philosophies associated with differing roles, distance, resources, organizational culture or priorities, other?

**RECOMMENDATIONS**

How could your organization better enable IKT?

Prompt

Same as above

**CONCLUSION**

Do you have any other suggestions for promoting or supporting IKT?
